# Adult Honeybees and Beeswax as Indicators of Trace Elements Pollution in a Vulnerable Environment: Distribution among Different Apicultural Compartments

**DOI:** 10.3390/molecules27196629

**Published:** 2022-10-06

**Authors:** Effrosyni Zafeiraki, Rastislav Sabo, Konstantinos M. Kasiotis, Kyriaki Machera, Lucia Sabová, Tomáš Majchrák

**Affiliations:** 1 Laboratory of Pesticides’ Toxicology, Department of Pesticides Control and Phytopharmacy, Benaki Phytopathological Institute, 145 61 Kifissia, Greece; 2 Department of Pharmacology and Toxicology, University of Veterinary Medicine and Pharmacy in Košice, Komenského 73, 041 81 Košice, Slovakia

**Keywords:** bioindicator, trace elements, ICP-MS, bees, honey, industry

## Abstract

Bees in search of diet sources intensively fly within a radius of up to 3 km, encountering nectar, pollen, and water sources which are potentially contaminated. Consequently, their products can provide valuable information about potential pollution. In the current study, 27 macro and trace elements, including the most hazardous ones, were measured in bees, honey, wax, pollen, and larvae, obtained from seven explicitly industrial areas in eastern regions of Slovakia, using a validated ICP-MS method. All the analysed elements were detected at least in one matrix. The detected concentrations of toxic elements, such as Hg, Pb, and Cd were in some cases higher in wax and bee samples, compared with honey, larvae, and pollen. In particular, Pb and Hg maximum concentrations were detected in the wax samples from Poša (3193 µg/kg) and Strážske_A (90 μg/kg). In addition, adult bees accumulated more elements than larvae, while wax and adult bees seemed more suitable for monitoring macro and trace elements in the surrounding environment. Statistical analysis emphasizing bees and wax correlated Cd with the Strážske area, possibly attributed to the intensified industrial activity in this region.

## 1. Introduction

Honeybees constitute an essential agriculture pillar, due to their fundamental role in pollination services. In addition, honeybees contribute to the food industry, as pollen and honey are edible apicultural products, well known for their exceptional nutritional value. However, it has been found that, apart from their content of lipid, proteins, bioactive compounds, and vitamins, etc., they also contain environmental contaminants, such as heavy metals, which honeybees accumulate and transfer to the beehives during the foraging period [1,2,3].

Concerns about the potential environmental and toxicological impact of toxic trace elements in honeybees and their products have oriented scientific interest towards the investigation of the occurrence of the former contaminants in bees and apiculture products [4,5,6,7,8,9,10,11,12]. Although metals including trace and macro elements are essential as nutrients for humans and also play a major role in many biochemical processes in living organisms at low levels [13], when they occur in higher quantities they can be toxic [14,15,16,17,18]. On the other hand, certain toxic trace elements and metalloids can provoke harmful toxic effects even at very low concentrations [19,20,21].

Sources of metal contamination in the environment are of both natural and anthropogenic origin. Increased concentrations of some metal elements are mainly due to human activities, e.g., industry, coal combustion, automobiles, and the burning of municipal waste [22]. According to several authors, honey seems to be a suitable indicator of the pollution of hazardous metals, since it reflects the toxic element content in the surrounding hive environment [8,9,23,24,25,26,27,28,29]. Some of these studies showed that different content of toxic elements detected in honey was mainly attributed to botanical origin, and not to the distance from the eventual source of pollution. But what about other relevant bee matrices? Can they interplay as suitable indicators for pollution analyses in areas with intensified industrial activity? This question primarily stems from the undisputable capacity of bees to cover a substantial geographical area as foragers. During this “trip,” bees come in contact with nectar, pollen and water sources, and therefore encounter a multitude of contaminants, including several potentially toxic elements. Bee larvae, the precedent of adult bees, are an important matrix both from an environmental perspective and from a toxicological aspect since they are more susceptible to deleterious chemical effects. The bee-larvae relationship is principally governed by insect metamorphosis, a physiological process often resulting in extreme changes in chemistry, morphology, and habitat. Changes in insect body chemistry during metamorphosis include the metabolization of body stores, the alteration of lipid and protein content, and the breaking down of specific cellular structures. From a toxicological point of view, it is a critical phase because contaminant concentrations and contaminant body burdens are also altered [30,31]. Finally, meconium also seems to be insects’ dominant excretory pathway in metal metabolization [32].

Overall, the aim of this study was to determine the content of both macro and trace elements in relevant bee matrices (honey, wax, pollen, bees, and larvae) sampled from explicitly industrial areas of the eastern part of Slovakia, and to set potential correlations between determined concentrations in different samples and apiculture matrices from the same sampling area. Through this strategy, the depiction of the potentially toxic trace elements and other elements’ status and variations among these industrialized areas was also contemplated.

## 2. Results and Discussion

### 2.1. Concentrations and Distribution of Elements

The determined concentrations of essential and toxic elements in relevant bee samples originating from industrial areas localized in the eastern part of Slovakia are given in Table 1, Table 2 and Table 3.

#### 2.1.1. Toxic Trace Elements

The concentration of As was detected in all the samples, with the highest concentration observed in wax (61 µg/kg) from Poša. A considerably high As concentration (54 µg/kg) was observed in bees from the locality of Prešov, possibly due to the presence of an active hazardous waste incineration plant in this city. The concentration of As detected in all the honey samples was <LOQ. As far as Hg detected levels were concerned, they were in almost the same range across all the analysed samples, with the highest concentrations of 90 and 36 µg/kg detected in wax samples both originating from the locality of Strážske. Generally, the concentrations of Hg detected in bees and wax seemed to be double, compared with the ones detected in larvae and honey. As regards Pb, an extensive variation in detected concentration was observed among the samples, with the highest concentration of 3193 µg/kg present in the wax sample from Poša. In addition, a considerably high Pb concentration (458 µg /kg) was detected in bees from the locality of Košice.

From honeys investigated in the current study, the highest concentration of Pb (47 µg/kg) was detected in the sample collected from Košice. Kováčik and colleagues [11] observed a concentration of Pb in the range of 7.36 ± 0.54 µg/kg in rapeseed honey and 85.6 ± 7.19 µg/kg in forest honey, what is comparable to the current findings. In the case of Cd, the highest concentration of 52.4 ± 3.52 µg/kg was found in forest honey, while two other samples contained 1.17 ± 0.18 µg/kg in black locust and 1.24 ± 0.26 µg/kg in rapeseed honey, which is in accordance with the findings of the present study, where Cd concentrations were <10 μg/kg. In the same context, Hg analysed in the honey sample originating from Košice showed a higher concentration (4.9 µg/kg) compared with the highest one of 2.65 ± 0.38 µg/kg detected in the forest honey, in a study carried out by Kováčik et al. (2016).

Considering the above, the current results are in agreement with the findings of Kováčik et al. (2016), indicating that the higher content of toxic metals detected in forest honey is most possibly attributed to the botanical origin of the sample. On the other hand, black locust and rapeseed honey analysed in a previous study are more similar to our honey samples in respect of botanical origin and time sampled. In another study, 34 mono- and multi-floral honey samples from four different geographical regions of Hungary were analysed for the detection of elements. According to the results, the maximum concentration of Pb was 133 µg/kg, while the highest concentrations of As and Cd were 30 and 3.3 µg/kg, respectively [10]. Recently, similar observations for Cd (<LOQ), Pb (44–81 µg/kg) and Hg not exceeding 5 µg/kg were also reported in Polish varietal honey [33].

#### 2.1.2. Other Trace Elements

A considerably high concentration of Sn was detected in wax samples originating from Poša and Strážske_B (3404 and 1304 µg/kg, respectively). Sb was detected in elevated concentrations in bees from Kurima and Košice (580 and 441 µg/kg, respectively); similarly, the pollen sample from Kurima contained very high levels of Sb (449 µg/kg). High Sb concentrations observed in the locality of Kurima could be explained by the past operation of an asphalt-producing plant in the area [34,35]. Although Ag levels were lower in honey and larvae compared with bees, no significant fluctuation of the detected Ag concentrations among the analysed samples was observed. On the other hand, a pronounced variation was observed in the concentration of Cd, mainly among the wax and bee samples, while in honey and larvae no Cd was detected, except for one sample of larvae from Kurima (with a concentration of 15 µg/kg). A high concentration of Ba was observed in all the pollen samples (1239–2884 µg/kg) and in some wax samples, with a maximum concentration of 9569 µg/kg detected in the locality of Poša. The concentrations of Tl and U were below LOQ in all the analysed samples, except for one wax sample from Strážske_B, in which the U concentration was equal to 11 μg/kg.

#### 2.1.3. Essential Elements

In general, narrow variation was observed among the concentrations of almost all the essential elements detected in the current samples (Table 3). The highest concentration of Na was detected in a bee sample (300 mg/kg) from Kurima. A higher concentration of Mg was detected in all the pollen samples (1086–1372 mg/kg) and in a wax sample from Strážske_B (1080 mg/kg). The concentrations of P and K were in almost the same range across all the samples, except for the wax samples, where a very wide variation of concentrations for P and K was observed (14–2949 mg/kg and 33–4013 mg/kg, respectively). The highest concentration of Ca was detected in a pollen sample from Sedliska (2377 mg/kg). The current levels were in accordance with values previously reported.

On the other hand, the Ca values obtained in the current honey samples were almost in the same range (167–209 mg/kg), but approximately 10 times higher compared with results (20.3 ± 3.09–36.6 ± 4.82 mg/kg) observed by Kováčik et al. (2016). The latter study showed a range of Na and Mg levels (8.49 ± 1.10–10.3 ± 1.52 mg/kg and 12.5 ± 2.44–65.0 ± 3.32 mg/kg, respectively,) detected in honey samples, while in the present study no Na and/or Mg was found in honey. The reason explaining the discrepancy in Ca, Na and Mg concentrations between the two studies is still unknown, and thus further research on this topic is needed. The reported concentrations of other dietary elements, such as K, Mg, Mn and Cu were in the same magnitude among the samples analysed in the two studies, while obtained concentrations of Fe and Cu were slightly higher compared with Kováčik et al. (2016).

Šedík et al. detected higher concentrations of all dietary (essential) elements in four different honey types (linden, sunflower, honeydew, and multifloral honey) collected from the vicinity of the city Nitra (in the western part of Slovakia) [36]. More specifically, linden honey contained the highest concentrations of Mn (2.7 mg/kg) and Zn (3.7 mg/kg), while multifloral honey contained only Ca (436 mg/kg). As far as the analysed honeydew honey sample is concerned, it contained the highest concentrations of several dietary elements: K (2687 mg/kg), Mg (121 mg/kg), Na (58 mg/kg), Fe (6.5 mg/kg) and Cu (2.8 mg/kg). The elevated levels of dietary elements detected in Šedík’s study compared with the current ones, could possibly be explained by the botanical origin of the honey samples; most of the elements detected in higher concentrations were present in linden and honeydew honeys.

The highest concentration of Cr was evidenced in the wax and bees samples, with the highest concentration detected in the wax sample originating from the locality of Poša (982 µg/kg); a substantially elevated concentration of 670 µg/kg was detected in larvae from the locality of Sedliska. Kováčik et al. (2016) analysed forest, black locust and rapeseed honey samples from the vicinity of the industrial town Košice (comparable to sampling area 6 of the present study) for the detection of selected metallic elements [11]. According to their findings, the highest concentration of Ni (1358.6 ± 241.9 µg/kg) was found in forest honey, while black locust and rapeseed honey contained 23.4 ± 3.81 µg/kg and 37.8 ± 5.26 µg/kg of Ni, respectively, in accordance with the findings of the present study.

The B concentrations are in almost the same range across the samples, with the highest concentration of 75,599 μg/kg detected in wax from Strážske_B. The same wax sample contained the highest concentrations of several elements such as Mn (41,904 μg/kg), Fe (160,775 μg/kg), Co (129 µg/kg), Ni (1919 µg/kg), and Zn (118,606 μg/kg) from all the wax samples analysed. A possible explanation of the high detected levels could be the age of the wax sample [37]. More specifically, this wax sample had the darkest colour compared to the other ones (brown colour).

As far as the current detected concentration of Cr in honey from Košice (42 µg/kg) is concerned, it was similar to the lowest levels found in black locust (26.8 ± 3.59 µg/kg) and rapeseed honey (43.3 ± 3.67 µg/kg analysed by Kováčik et al. (2016).

A very high concentration of Cu was observed in pollen and bees (15,994 µg/kg and 15,729 µg/kg, respectively) in Košice, maybe due to the presence of the steel industry in this city.

In a study focusing on the analysis of 30 honey samples of two different types (rapeseed and honeydew) and collected from various locations within Poland, the reported levels of Mn (7.37 ± 0.20 mg/kg), Ni (1.326 ± 0.011 mg/kg), K (3659.3 ± 1.9 mg/kg), Fe (16.1 ± 0.3 mg/kg), Cu (1.82 ± 0.09 mg/kg) and Zn (9.93 ± 0.21 mg/kg) in honeydew honey samples were higher, compared with the current results. In contrast, the highest levels of B (15.9 ± 0.14 mg/kg) and Ca (159.2 ± 2.23 mg/kg) detected in rapeseed honey samples were in the same range in both studies. It is worth mentioning that the concentrations of the former elements (Al, Mn, Ni, K, Fe and Zn) detected in rapeseed honey samples were in the same range as the ones detected in the present study, except for the concentration of Na (89.60 ± 0.36 mg/kg) and Mg (19.83 ± 0.82 mg/kg), which were higher, compared with the latter [8].

The concentrations of metal elements (B, Cu, Mg, Ni and Zn) detected in Czech flower honey samples harvested in 2003 and 2004 were in concordance with the current findings. Only the maximum element concentrations of Ca and Mn (142.0 and 4.92 mg/kg) were lower and higher, respectively, compared with our findings [38].

### 2.2. Honeybees Results Compared to Pb, Cr, Ni, and Cd Reference Values

For honeybees, the determination of low and high threshold values for Pb, Cr, Ni, and Cd [39] used as a metric of metal contamination of terrestrial areas [40,41] prompted us to proceed to straightforward comparisons. More specifically, exceedances of 700, 120, 300, and 100 μg/kg for Pb, Cr, Ni and Cd, respectively, indicate high-pollution areas. Respective lower limits are set at 300, 40, 100 and 52 μg/kg for Pb, Cr, Ni and Cd, correspondingly. Therefore, based on the presented results in bees (all calculated on wet matter, in line with the threshold values also calculated on a wet weight basis) for Cr, all areas indicated high pollution, with the exception of Strážske_A. For Ni, in the Strážske_B and Prešov localities only, values were below the worrisome level, yet worthy of attention; the rest of the values surpassed the upper limit. A lower pollution picture was produced when Cd and Pb were introduced in respective comparisons. As regards Pb, no exceedance was observed, while Cd levels surpassed the upper limit in the Strážske_A and Prešov localities. Overall, the presented contradistinction to a large extent verifies the intermediate to highly polluted toxic trace elements profile of the respective areas.

### 2.3. Correlations of Selected Elements

To determine the correlation between the concentrations detected among the different matrices originating from the same sampling area was a challenging subject. To our knowledge, no paper dealing with this approach is available, and so we could only marginally compare our findings with the literature. This comparison focused on the correlation between bees and honey or pollen, between larvae and honey or pollen, and finally on the correlation between bees and larvae from the same sampling area. Since some concentrations were below the LOQ (Table 1), correlation values (CVs) were not determined for all the metals. The CVs determined for the detected trace and macro elements (concentrations > LOQ) are presented in the Supplementary Data (Tables S1 and S2).

With regard to Hg, the CV among the determined concentrations is almost in the same range across the samples (Supplementary Data). A very high CV of 24.01 between the Cr concentrations determined in bees/honey was observed in sampling area Strážske_B. In contrast to this, in sampling area Strážske_A this value was only 1.97. The highest correlation values of 26.16 and 18.49 between determined concentration of Ni in larvae/honey and in bees/honey, respectively, was detected in sampling area 3 (Poša). It was also difficult to obtain a general pattern for Pb because determined CVs are wide, with the highest value of 9.86 in bees/honey from area 5 (Prešov).

Generally, the determined CVs between analysed concentrations of P, K and Ca in bees/honey and larvae/honey were within the same range but higher compared to the bees/pollen, larvae/pollen and bees/larvae ratio, except for the higher bees/larvae ratio of 4.07 determined in Košice.

Lipids and proteins are the main constituents of royal jelly [41] derived from pollen [42]. The fate of elements found in lipophilic matrices as wax originating from pollen and honey during this process remains unknown. However, based on the current detected levels in larvae, it could be assumed that these elements are present in the larval diet. The fat body in bee larvae is considered as a key organ, and a major components of the fat body and the main source of metabolic fuel, are lipids [43].

Most of the observed CVs in the bees/larvae ratio across the sampling areas are higher than one (except for some essential elements; please see Supplementary Data), indicating a higher cumulative capacity of adult bees compared with their larval stage. This finding opposes the literature [32]. Kraus and colleagues analysed data from 39 studies of 68 analytes (metal elements included). They observed that metals were predominantly lost during metamorphosis, leading to ∼2 to 125-fold higher larval concentrations. The honeybees belong to holometabolous insects, which means they undergo egg, larva, pupa, and adult phases, while during their larval development phase five instars are recognized. There are two main routes of metabolization of the contaminant by insects—via exuvia (exoskeleton) shed by final larval instar and via meconium excretion, while the second seems to play a dominant role in metal metabolization [30,32].

According to the results of the current study, the concentrations of the analysed essential and toxic elements in pollen and honey samples can be considered as “a matter of chance with random character”, and depend strongly on the time of sampling, i.e., during or just before our sampling time foragers collected pollen and/or honey of various botanical origins with different metal element contents (Table 1, Table 2 and Table 3).

To link pollen to its botanical origin is laborious work; moreover, to determine the exact origin of honey is impossible, due to missing validated methods and laboratory techniques. That is why the analysis of metal element concentrations in pollen and honey is more supportive without this linking and has a more or less informative character in searching for correlations among determined concentrations. Overall, and since it is beyond the scope of the presented work to proceed to nectar isolation-collection (from plants) and the subsequent ICP-MS analysis, it is risky to correlate findings with the plant source, even though beekeepers’ observations concerning bees’ visits were considered. Nevertheless, the presented results postulate that intensified industrial activity, documented in the sampling areas, is a key driver of the observed concentrations of toxic trace elements in apiculture matrices.

Beeswax serves as a detoxication “colony organ” for various lipophilic xenobiotics present in the flying radius of foragers, a fact confirmed by our results (Table 1, Table 2 and Table 3). In this study some concentrations analysed in beeswax samples were much higher, compared with bees and/or larvae, or at least equal (e.g., Hg, Pb and others), thus making this type of sample more suitable for the analysis of potential metal pollution in the environment. Moreover, comparing results of metal element concentrations in wax samples from the same sampling locality of Strážske (the first two sampling areas) confirmed the necessity to regularly exchange wax in apiculture. Detected higher levels of toxic trace elements in very old wax originating from sampling area 2 (Strážske_B) can have a potentially negative impact on health status, not only on the bee colony, but also on humans as consumers (the potential transfer of toxic trace elements to honey and/or bee bread).

When the ratios of concentrations of essential and hazardous metal elements between adult bees and larvae were compared, adult bee bodies seemed to contain higher concentrations of metal elements. Observed concentrations of toxic trace elements, e.g., Hg, Pb, and others, were in some specific cases several times higher in adult bees than in larvae (please see Supplementary Data), thus making them more suitable for analysis.

Based on the observation of a higher metal element content in most samples obtained from sampling area 1 (Strážske_A) compared with the levels from sampling area 2 (Strážske_B) with 1.5 km distance between them, we can infer that the distance from the eventual source of pollution seems to play an important role in analyses results of potential metal pollution on the environment (Table 1, Table 2 and Table 3).

### 2.4. Statistical Analysis Results

The implementation of four normality tests verified the normal distribution of the concentration values for Hg in bees and larvae (for Hg, see Figure S1A,B depicting quantile-quantile (Q-Q) plots, and statistical values in Table S3). On the contrary, Pb respective values do not follow the normal distribution, with the exception of the Jarque-Bera test consideration (Table S4). As an additional step, a t-test and z-test were applied. For these tests, the limited number of replicates per sampling area directed us to focus on the matrices, rather than including the sampling location as a statistical parameter. Hence, for all trace and macro elements, statistical analysis assessed the significance of differential element concentrations obtained among relevant matrices, epitomized by the bees/larvae comparison. The overall results showed that the latter was element-dependent, exhibiting both significant and non-significant values.

**Figure 1:**
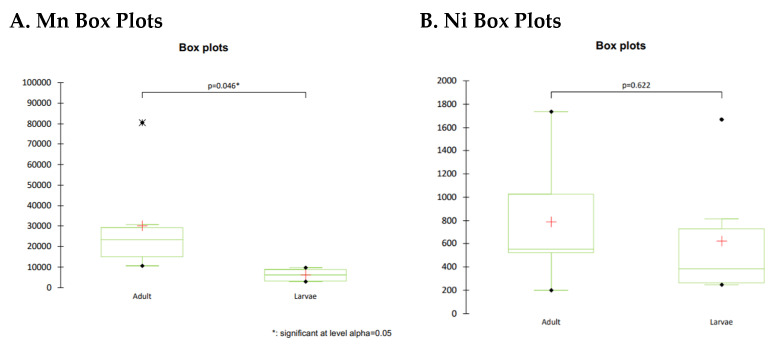
Exemplary box plots for Mn (**A**) and Ni (**B**), comparing adult bees with larvae.

Indicatively, for Mn levels in bees and larvae, the respective box plots are provided in Figure 1A. As the computed *p*-value is lower than the significance level alpha = 0.05, one should reject the null hypothesis H0, and accept the alternative hypothesis Ha.

Hence, for Mn there is statistical significance in the observed results. On the contrary, for Ni (Figure 1B), the absence of a definite trend in obtained concentrations leads to non-statistical significance (*p*-value > 0.05). One-way ANOVA also produced the same outcome for all respective comparisons.

To further exploit the results and incorporate the areas in the subsequent discussion, a principal component analysis (PCA) was implemented. PCA was conducted for all matrices as regards the toxic trace elements (Hg, As, Cd, Pb). Emphasis was given to bees and wax since, based on the findings, they seemed to be superior bioindicators compared with the rest of the apiculture matrices.

**Figure 2:**
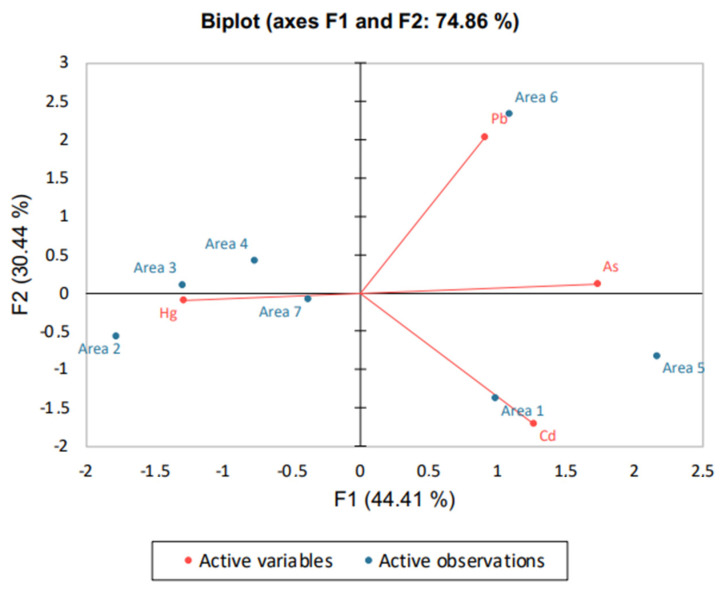
PCA biplot produced for toxic trace elements on bees.

When PCA was applied to toxic trace elements in bees (see PCA biplot in Figure 2, for scree plot of eigenvalues and % cumulative variability see Figure S2, and for eigenvectors, factor loadings, correlations and squared cosines advise Table S5), Cd was associated with area 1 (Strážske), Hg with area 3 (Poša) and Pb with area 6 (Košice).

**Figure 3:**
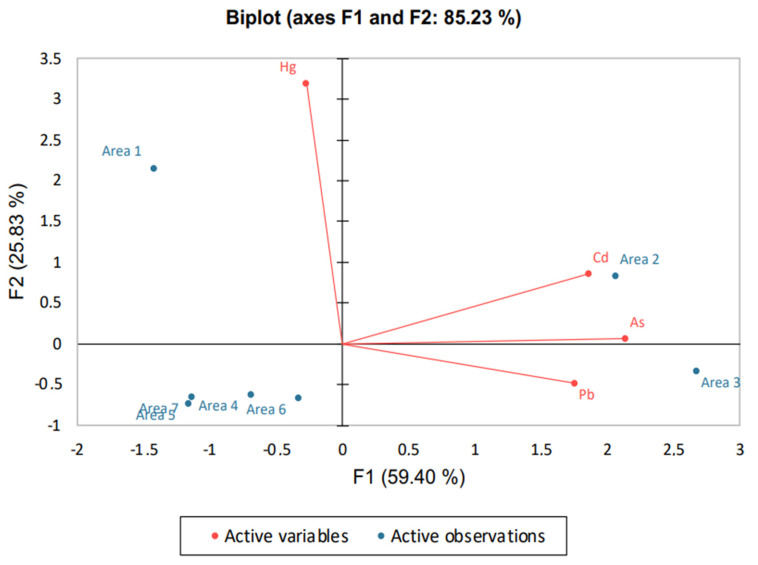
PCA biplot produced for toxic trace elements on wax.

Nevertheless, the PCA outcome and subsequent interpretations are also matrix-dependent. When PCA was applied to toxic trace elements in wax, a different picture was produced (see Figure 3), exhibiting only a strong correlation of Cd with area 2. Cd preponderance in areas 1 and 2 can be attributed to the intensive military-industrial activity and processes in which Cd can be a constituent or intermediate product. Previous publications manifested elevated levels of toxic trace elements (including Cd) in army shooting range areas, due to ammunition [44,45]. Overall, the above data indicate the different accumulation character of some elements in relation to the studied matrix, but also highlight the multiple routes of exposure to which adult bees especially are subjected. Last but not least, in all PCAs conducted, the first two components (or factors, F) explained more than 70% of the total variance in initial values.

## 3. Materials and Methods

### 3.1. Short Description of Sampling Areas

Sampling was focused on seven industrialized areas in eastern Slovakia, aspiring to investigate “the worst case” scenario for the bee colonies and consumers. More specifically, sampling areas 1 and 2 are both in the cadastre of the town Strážske, where a chemical producing plant has been active for a long time, since 1952. Until 2003, the plant was focused on the production of explosives and other intermediate products intended for both military and civilian uses. Sampling area 1 is closer to this plant. Area 3 (village Poša) is near the basin where the contaminated wastewater channel from the chemical producing plant in Strážske ends (6 km to the west of this plant) and 2.5 km to the south of the active plant focused on wood mass chemical processing (near the city of Vranov nad Topľou). Sampling area 4 (village Sedliska) is localized 5 km to the north of the plant focused on wood mass chemical processing and 7 km to the northwest of the chemical producing plant in Strážske. Area 5 (city Prešov) has an active hazardous waste incineration plant, while area 6 (city Košice) has a long-time active steel factory and waste incineration plant. Finally, sampling area 7 (village Kurima) is localized near an asphalt-producing plant that was long time active in the past. Therefore, six heavily industrialized areas (some of them with still active plants) and one historic industrialized area (area 7) were chosen for this project.

### 3.2. Sampling

**Figure 4:**
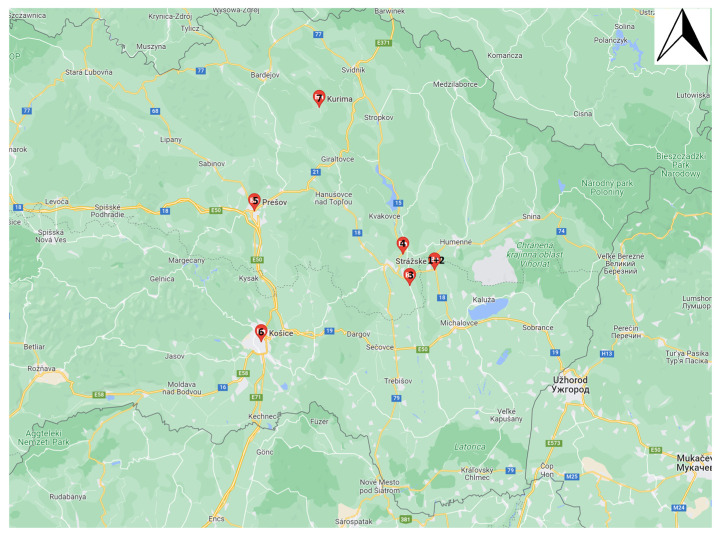
Map with sampling regions in Slovakia.

For the purpose of the current study, honey, wax, pollen, bees and larvae were collected between 18 May 2020 and 23 May 2020 from six heavily industrialized areas and one historic industrialized area in eastern Slovakia (Figure 4). Each sample was a pool sample originating from three beehives from the same locality. Only beekeepers from areas 4, 6 and 7 could provide pooled pollen collected on three hive entrances (using a pollen trap). The total weight of the larvae sample from locality 2 was insufficient, since only young larvae were collected from three swarms/nuclei (old larvae were not present at that time). Honey sample from sampling area 7 was not analyzed, since it was damaged during its transport to the laboratory. The samples of honey were collected from frames inserted into hives two weeks prior to sampling, in order to avoid the old winter hive stores. In contrast, the collection of wax samples was focused on the oldest ones (dark colour). The same was applied to the larvae; the older larvae were preferred by the embraced sampling, as the absolute amounts of lipids increase in worker larvae as they increase in weight during their larval development, and, in contrast, the amounts decrease during metamorphosis [46].

Bees were sampled from the brood combs from three hives per sampling area. Detailed analyses of pollen and honey origin were not performed, as it was not the aim of this study. Nevertheless, honey samples 1–4 were predominantly oilseed rape (OSR) honeys. This statement was supported by details provided by experienced beekeepers, based on visual observations of flowering crops carried out in the vicinity of the apiary (2–3 km) before sampling and on daily inspection of foragers’ flight direction and of the honey harvest performed later (oilseed rape honey has a typical character, and is easily visually detectable). Samples 5–6 were multifloral honeys, since respective areas are city apiaries with multifloral sources of nectar/pollen (parks, avenues, gardens, etc.). None of the honey samples was honeydew; honeydew occurs in late summer and/or autumn and not in late spring. Lastly, area 7 is in the countryside without any flowering OSR in a radius of 3 km from the apiary at or before the time of sampling.

All samples were stored in the freezer at −18 °C and sent from Slovakia to Greece under cold conditions (0–4 °C) within 2 days, where they were kept again at −18 °C, prior to treatment.

### 3.3. Chemicals

The ICP-MS method of analysis used is suitable for the quantitative determination of 27 chemical elements: Boron (B), Magnesium (Mg), Aluminium (Al), Vanadium (V), Chromium (Cr), Manganese (Mn), Iron (Fe), Cobalt (Co), Nickel (Ni), Copper (Cu), Zinc (Zn), Arsenic (As), Strontium (Sr), Molybdenum (Mo), Silver (Ag), Cadmium (Cd), Tin (Sn), Antimony (Sb), Barium (Ba), Mercury (Hg), Thallium (Tl), Lead (Pb), Uranium (U), Sodium (Na), Phosphorus (P), Potassium (K) and Calcium (Ca). Standard solutions of 25 chemical elements mix (Al, Ba, B, Cu, Fe, Sr, Zn, Be, Cr, Co, Li, Mn, Mo, Ni, Ti, V, Sb, As, Cd, Pb, Se, Ag, Tl, Sn, U in HNO_3_ 5%) and of individual elements (Hg in 10% HNO_3_, P in 0,05% H_2_SO_4_ and Na, Mg, and Ca in 2% HNO_3_), used for the preparation of two calibration curves, were all purchased by CPAchem (Stara Zagora, Bulgaria). Individual solutions of Lithium (Li), Scandium (Sc), Germanium (Ge), Yttrium (Y), Indium (In), Terbium (Tb) and Iridium (Ir) used as internal standards and a standard solution/mixture of 25 element components (Al, Be, Co, Li, Se, Sn, Zn, Sb, B, Cu, Mn, Ag, Ti, As, Cd, Fe, Mo, Sr, U, Ba, Cr, Pb, Ni, Tl, V in HNO_3_ 5%) used for the preparation of a quality assurance (QC) standard, were also provided by CPAchem (Stara Zagora, Bulgaria). The two CRMs, BCR-191 brown bread and BCR-679 white cabbage, were provided by the European Commission, while hydrogen peroxide 30% *w*/*w* (H_2_O_2_) and nitric acid (HNO_3_) 67–69% *w*/*w* for trace elements analysis were all purchased by Seastar Chemicals Inc. (Sidney, Canada). Moreover, ultrapure water from an ultrapure Milli-Q water system was used for the dilution of the aforementioned solutions, when needed. Prior to the analysis of the samples, the labware used was immersed in a diluted HNO_3_ solution (0.05% *w*/*w*) with ultrapure water for 24 h, washed several times with ultrapure water, and finally dried inside a hood under clean-air conditions.

### 3.4. Chemical Analysis

#### 3.4.1. Sample Preparation

Upon arrival at the laboratory, pollen samples were separated and left to dry (30 h) inside a laboratory hood under ambient temperature. The rest of the matrices were used intact. Subsequently, 0.5 g of each sample (all commodities) were weighted and placed into a vessel in order to be further digested in a microwave oven (ETHOS UP, Milestone Srl, Italy). Subsequently, 9 mL HNO_3_ and 1 mL H_2_O_2_ were added to each vessel and the samples were digested under 180 °C and 800 Watt for 40 min total. After the end of the digestion process, the vessels were cooled to less than 40 °C in a clean hood. The digestion solution was further transferred into a clean container and diluted with ultrapure water to a final volume of 100 mL.

#### 3.4.2. Instrumental Analysis

All the samples were analysed using an ICP-MS (Thermo iCAP-RQ, Thermo Fisher Scientific, San Jose, CA, USA), equipped with an ASX-280 autosampler, as described in detail in previous publications of our analytical group [47]. Briefly, for the quantification of the 27 macro and trace elements, Ni sample and skimmer cones, a Quartz cyclonic spray chamber and a MicroMist U-Series Nebulizer (0.4 mL/min with PEEK connector) were used. The flow of the nebulizer gas and the cool gas was approximately 1 L/min (autotune dependent) and 14 L/min, respectively. Each batch of samples was analyzed in KED mode, while a performance report in KED mode was also conducted prior to each analysis, in order to assure the high sensitivity and stability of the measurement. In this context, the occurrence of interferences and doubly charged ions were also minimized.

#### 3.4.3. Qualification and Quality Assurance

Calibration curves (five points including zero) were prepared to match the expected concentration ranges of trace and macro elements in the samples. More specifically, calibration curves covering concentrations between 0.1 and 1000 ppb and others covering concentrations from 0.1 to 200 ppm were used for the quantification of trace and macro elements, respectively. The linear regression (r^2^) was greater than 0.999 for all elements in the corresponding calibration curves. Gold (Au) was also added in all the calibration standards for the stabilization of mercury.

The limits of quantification (LOQ) values for each individual element are presented in Table 4. In order to monitor and assure the accuracy and repeatability of the measurements, two quality-control (QC) standard solutions of macro and trace elements and a Certified Reference Material (CRM), BCR-679 white cabbage and spiked sample (one of each matrix, following the same procedure with the unknown samples) were analysed in every batch of samples. Seven internal standards (6 Li, Sc, Ge, Y, In, Tb and Ir) were also added to all unknown samples, at a constant rate. The recovery of the QCs and the internal standards ranged between 80–120% in all the analysed samples. The recoveries for the elements contained in the CRM (Cd, Cu, Fe, Mn, Mo, Ni, Sr, Zn, Hg, Sb, Tl, B, Ba, Ca, Cr, Mg, P) were also in the range of 80–120%, and thus no correction of the analytical data presented was needed. To further verify the accuracy of the method, the CRM and two unknown samples of each matrix were analysed by another laboratory. The analysis in both laboratories was conducted using a microwave digestion system and ICP-MS. There was no significant difference between the obtained results of the two laboratories.

For the avoidance of spectral interferences, reagent blanks were also analysed, followed the same procedure with the unknown samples and measured in each batch, while one additional isotope was measured when possible.

### 3.5. Statistical Analysis

Four normality tests were applied (Shapiro-Wilk, Anderson-Darling, Lilliefors and Jarque-Bera) to the set of concentration values of toxic trace elements, for which more than five >LOQ values were obtained. These cases concerned Hg and Pb. If the computed *p*-value is greater than the significance level alpha = 0.05, one cannot reject the null hypothesis, meaning, the variable from which the sample was extracted follows a normal distribution. Additionally, the student’s t-test on two independent samples and z-test were applied to assess the statistical significance of the macro and trace elements concentrations found among the studied matrices. The latter was exemplified by the comparison of element levels in bees and larvae. Sampling area Strážske_B was excluded from these calculations since the larvae were not analysed to provide respective values. One-way analysis of variance (ANOVA) was also applied to the same dataset. Subsequently, principal component analysis (PCA) was also attempted, focusing on toxic trace elements (Hg, As, Cd, Pb). To proceed with an appropriate dataset for PCA, for each matrix the trace elements were considered as the variables and the locations were the observations. Standardization of values was implemented by subtracting the mean value, then dividing by the standard deviation. As stated above, the significance level alpha = 0.05 was considered. Statistical analysis was conducted using XLSTAT.

## 4. Conclusions

Considering the persistent character and hazardous potential of some toxic trace elements, it is important to include essential and toxic elements in targeted analytical methodologies. Our results confirmed the finding that the content of hazardous elements detected in honey is dependent on the honey origin; findings were comparable with the literature. Moreover, the presented results show that wax and adult bees are both more suitable for monitoring and analysis of macro and trace elements in the surrounding environment. Observed concentrations (and average values) of toxic trace elements, e.g., Hg, Pb, Cd and other elements such as Ag, Cr and Sn were in some specific cases several times higher in wax and/or bee samples, compared with larvae, honey or pollen samples, thus making them more suitable for metal content analysis in the environment. A higher cumulative capacity of metal elements in adult bees compared with their larval stage was also corroborated. Furthermore, these findings could help disclose the fate of elements originating from pollen and honey in the diet and life circle of the bee colony in the future.

## Figures and Tables

**Table 1:** Content (µg/kg) of toxic trace elements.

Sample	Sampling Location	As	Hg	Pb	Cd
**WAX**	1. Strážske_A	<LOQ	90	59	<LOQ
	2. Strážske_B	59	36	589	60
	3. Poša	61	24	3193	29
	4. Sedliska	17	15	174	<LOQ
	5. Prešov	<LOQ	12	79	<LOQ
	6. Košice	23	16	604	<LOQ
	7. Kurima	<LOQ	15	138	<LOQ
**BEES**	1. Strážske_A	17	11	101	161
	2. Strážske_B	13	27	37	56
	3. Poša	<LOQ	13	66	20
	4. Sedliska	12	15	130	26
	5. Prešov	54	11	115	131
	6. Košice	33	13	458	27
	7. Kurima	13	11	65	38
**POLLEN**	4. Sedliska	<LOQ	12	86	33
	6. Košice	29	10	226	66
	7. Kurima	13	9.2	75	152
**HONEY**	1. Strážske_A	<LOQ	7.0	43	<LOQ
	2. Strážske_B	<LOQ	6.4	<LOQ	<LOQ
	3. Poša	<LOQ	5.9	<LOQ	<LOQ
	4. Sedliska	<LOQ	6.2	<LOQ	<LOQ
	5. Prešov	<LOQ	5.4	12	<LOQ
	6. Košice	<LOQ	4.9	47	<LOQ
**LARVAE**	1. Strážske_A	<LOQ	5.2	28	<LOQ
	3. Poša	<LOQ	7.7	31	<LOQ
	4. Sedliska	24	8.0	47	<LOQ
	5. Prešov	<LOQ	5.4	30	<LOQ
	6. Košice	<LOQ	6.1	49	<LOQ
	7. Kurima	<LOQ	8.2	31	15

**Table 2:** Content (µg/kg) of other trace elements.

Sample	Sampling Location	Al	Ba	Sb	Sn	Ag	Sr	V	Tl	U
**WAX**	1. Strážske_A	5853	<LOQ	173	103	65	<LOQ	<LOQ	<LOQ	<LOQ
	2. Strážske_B	132,234	4135	49	1304	42	3313	235	<LOQ	11
	3. Poša	114,352	9569	59	3404	21	1572	198	<LOQ	<LOQ
	4. Sedliska	41,255	778	28	74	14	<LOQ	74	<LOQ	<LOQ
	5. Prešov	9950	333	44	97	17	<LOQ	<LOQ	<LOQ	<LOQ
	6. Košice	54,276	2942	45	209	16	710	113	<LOQ	<LOQ
	7. Kurima	19,368	534	21	64	31	<LOQ	31	<LOQ	<LOQ
**BEES**	1. Strážske_A	13,851	636	22	25	<LOQ	730	<LOQ	<LOQ	<LOQ
	2. Strážske_B	8561	283	19	52	<LOQ	<LOQ	<LOQ	<LOQ	<LOQ
	3. Poša	8912	472	44	34	57	663	34	<LOQ	<LOQ
	4. Sedliska	18,111	526	84	55	32	712	25	<LOQ	<LOQ
	5. Prešov	13,587	368	80	11	14	596	<LOQ	<LOQ	<LOQ
	6. Košice	49,926	1422	441	113	99	1104	109	<LOQ	<LOQ
	7. Kurima	12,800	803	580	23	38	552	26	<LOQ	<LOQ
**POLLEN**	4. Sedliska	27,773	1239	23	25	76	2196	53	<LOQ	<LOQ
	6. Košice	59,677	2400	28	40	14	1222	134	<LOQ	<LOQ
	7. Kurima	33,821	2884	449	35	75	1566	67	<LOQ	<LOQ
**HONEY**	1. Strážske_A	6472	241	49	15	14	<LOQ	<LOQ	<LOQ	<LOQ
	2. Strážske_B	4164	<LOQ	18	5.1	<LOQ	<LOQ	27	<LOQ	<LOQ
	3. Poša	6464	<LOQ	31	7.7	11	<LOQ	<LOQ	<LOQ	<LOQ
	4. Sedliska	3589	<LOQ	19	4.2	<LOQ	<LOQ	<LOQ	<LOQ	<LOQ
	5. Prešov	3926	<LOQ	17	14	<LOQ	<LOQ	<LOQ	<LOQ	<LOQ
	6. Košice	5683	<LOQ	17	9.4	10	<LOQ	<LOQ	<LOQ	<LOQ
**LARVAE**	1. Strážske_A	7695	300	6	8.7	<LOQ	<LOQ	<LOQ	<LOQ	<LOQ
	3. Poša	6360	303	17	12	<LOQ	<LOQ	<LOQ	<LOQ	<LOQ
	4. Sedliska	10,313	364	24	89	12	<LOQ	<LOQ	<LOQ	<LOQ
	5. Prešov	6695	<LOQ	10	24	<LOQ	<LOQ	<LOQ	<LOQ	<LOQ
	6. Košice	4700	<LOQ	11	24	<LOQ	<LOQ	<LOQ	<LOQ	<LOQ
	7. Kurima	10,856	542	10	19	35	<LOQ	27	<LOQ	<LOQ

**Table 3:** Content of essential macro (mg/kg) and trace (µg/kg) elements.

				mg/kg						µg/kg					
Sample	Sampling Location	Na	Mg	P	K	Ca	Cu	Fe	Mn	Zn	Cr	B	Ni	Mo	Co
**WAX**	1. Strážske_A	<LOQ	<LOQ	14	33	129	280	8277	182	5150	122	1196	438	191	<LOQ
	2. Strážske_B	118	1080	2949	4013	1427	12,967	160,775	41,904	118,606	382	75,599	1919	438	129
	3. Poša	132	129	123	599	632	4244	127,048	4077	31,538	982	8675	397	102	84
	4. Sedliska	<LOQ	213	407	1159	376	751	34,065	2531	8244	257	22,041	205	44	<LOQ
	5. Prešov	<LOQ	<LOQ	63	170	168	<LOQ	12,037	370	4037	196	3206	94	25	<LOQ
	6. Košice	<LOQ	407	542	1924	701	1033	92,325	2970	11,425	287	24,667	243	48	29
	7. Kurima	<LOQ	116	105	361	326	290	29,411	904	5959	82	5971	136	<LOQ	<LOQ
**BEES**	1. Strážske_A	221	354	2046	2793	597	8752	42,476	22,392	25,396	85	15,070	515	185	<LOQ
	2. Strážske_B	107	240	1360	1957	314	4991	40,368	16,366	16,732	830	10,310	299	182	101
	3. Poša	225	420	2244	3028	475	13,127	33,039	10,649	44,597	199	22,476	1177	148	26
	4. Sedliska	222	469	2502	3665	721	7005	58,972	24,041	29,051	173	21,008	539	136	67
	5. Prešov	152	425	2547	3834	513	8782	101,015	80,468	39,908	153	10,452	197	130	65
	6. Košice	248	671	3458	5454	1050	15,729	130,497	30,859	48,656	623	9084	1735	402	95
	7. Kurima	300	626	4107	6396	590	12,624	56,724	12,601	53,819	158	9566	572	111	41
**POLLEN**	4. Sedliska	<LOQ	1342	4670	5902	2377	7819	51,642	32,979	32,072	112	98,905	1422	498	40
	6. Košice	<LOQ	1372	5539	7984	2238	15,994	145,701	41,652	70,121	305	47,864	1124	388	118
	7. Kurima	<LOQ	1086	4269	7383	1395	9394	65,320	53,103	40,129	129	26,425	3367	145	83
**HONEY**	1. Strážske_A	<LOQ	<LOQ	52	293	209	272	4824	642	1099	43	11,292	112	<LOQ	<LOQ
	2. Strážske_B	<LOQ	<LOQ	42	168	173	<LOQ	1258	244	363	35	14,185	56	<LOQ	<LOQ
	3. Poša	<LOQ	<LOQ	30	163	167	<LOQ	2882	330	282	52	17,574	64	<LOQ	<LOQ
	4. Sedliska	<LOQ	<LOQ	42	633	186	270	1424	710	478	38	15,698	68	<LOQ	<LOQ
	5. Prešov	<LOQ	<LOQ	46	777	187	<LOQ	1856	540	1073	42	11,424	42	<LOQ	<LOQ
	6. Košice	<LOQ	<LOQ	45	1265	199	553	2563	671	1051	42	6082	104	<LOQ	<LOQ
**LARVAE**	1. Strážske_A	187	800	4895	8167	516	12,291	28,911	8835	46,288	83	18,653	459	184	<LOQ
	3. Poša	208	737	4565	7489	362	11,364	25,306	2839	45,956	120	20,067	1665	164	<LOQ
	4. Sedliska	263	849	5101	9480	560	12,599	1,605,132	9786	75,536	670	28,259	816	167	29
	5. Prešov	232	704	3924	7396	332	10,284	33,345	3873	39,145	89	12,042	249	138	<LOQ
	6. Košice	167	621	3393	6844	258	8155	25,321	2751	38,686	70	7738	314	148	<LOQ
	7. Kurima	146	652	4056	7805	368	9610	28,533	8290	49,258	54	10,160	248	144	<LOQ

**Table 4:** Quantification parameters of trace and macro elements.

Element	Symbol	Reporting Isotope	Interference Correction	ISTD	LOQ	Unit	Expanded Uncertainty. U (%) *
Boron	Β	11		^6^Li	500	µg/kg	11.48
Sodium	Na	23		^45^Sc	100	mg/kg	11.94
Magnesium	Mg	24		^45^Sc	100	mg/kg	8.94
Aluminium	Al	27		^45^Sc	250	µg/kg	10.46
Phosphorus	P	31		^45^Sc	1	mg/kg	9.92
Potassium	K	39		^45^Sc	1	mg/kg	7.00
Calcium	Ca	44		^45^Sc	100	mg/kg	8.34
Vanadium	V	51		^45^Sc	25	µg/kg	11.10
Chromium	Cr	52		^72^Ge	25	µg/kg	14.34
Manganese	Mn	55		^72^Ge	100	µg/kg	15.10
Iron	Fe	56		^72^Ge	250	µg/kg	5.96
Cobalt	Co	59		^72^Ge	25	µg/kg	13.12
Nickel	Ni	60		^72^Ge	25	µg/kg	10.04
Copper	Cu	63		^72^Ge	250	µg/kg	5.44
Zinc	Zn	66	64Zn: −0.0348659*60Ni (KED)	^72^Ge	250	µg/kg	18.72
Arsenic	As	75		^72^Ge	10	µg/kg	8.54
Strontium	Sr	88	86Sr: −1.50435*83Kr (KED)	^89^Υ	500	µg/kg	17.28
Molybdenum	Mo	95		^89^Υ	25	µg/kg	12.18
Silver	Ag	107		^115^In	10	µg/kg	10.64
Cadmium	Cd	111	114Cd: −0.0268373*118Sn (KED)	^115^In	10	µg/kg	9.92
Tin	Sn	118		^115^In	1	µg/kg	10.12
Antimony	Sb	121		^159^Tb	1	µg/kg	19.36
Barium	Ba	137		^159^Tb	250	µg/kg	18.26
Mercury	Hg	202		^191^Ir	2	µg/kg	6.76
Thallium	Tl	205		^191^Ir	10	µg/kg	8.52
Lead	Pb	208	208Pb: 1*207Pb (KED) + 1*206Pb (KED)	^191^Ir	10	µg/kg	10.24
Uranium	U	238		^191^Ir	10	µg/kg	10.12
**Internal standards**	**Symbol**	**Reporting isotope**	**Interference correction**				
	Li	6					
	Sc	45					
	Ge	73					
	Y	89					
	In	115	−0.0148637*118Sn (KED)				
	Tb	159					
	Ir	191					

* Uncertainty (expanded) is calculated at the level of 10LOQ and the level of confidence is 95%.

## Data Availability

All data available are presented in this manuscript.

## Supplementary Materials

The following supporting information can be downloaded at: https://www.mdpi.com/article/10.3390/molecules27196629/s1, Table S1: Correlation values between relevant bee samples (essential and toxic trace elements); Table S2: Correlation values between relevant bee samples (essential macro elements); Table S3: Overview of computed *p*-values for Hg in Bees and Larvae; Table S4: Overview of computed *p*-values for Pb in Bees and Larvae; Table S5: Eigenvectors, factor loadings, correlations between variables and factors, and squared cosines of the observations, for toxic trace elements on bees; Figure S1: Quantile-quantile plots for Hg concentration values in bees and larvae; Figure S2: Scree plot of eigenvalue and % cumulative capacity for toxic trace elements on bees.

## Sample Availability

No sample availability.
