# Supplementary material for: Adult Honeybees and Beeswax as Indicators of Trace Elements Pollution in a Vulnerable Environment: Distribution among Different Apicultural Compartments
Source: Molecules. 2022 Oct 6;27(19):6629. doi: 10.3390/molecules27196629 (PMC9572923; doi:10.3390/molecules27196629)
Supplement: Supplementary file 1 [file molecules-27-06629-s001.zip › molecules-1913028-supplementary.pdf]

Honeybees and their products as bioindicators of heavy metals pollution in a vulnerable environment: distribution among different apicultural compartments

Effrosyni Zafeiraki,[1,*] Rastislav Sabo,[2,] Konstantinos M. Kasiotis,[1,*] Kyriaki Machera,[1] Lucia Sabová,[2] Tomáš Majchrák[2]

Table S1: Correlation values between relevant bee samples (essential and toxic trace elements)

Sample	sampling location	B	Al	V	Cr	Mn	Fe	Co	Ni	Cu	Zn	As	Sr	Mo	Ag	Cd	Sn	Sb	Ba	Hg	Pb
**bees/ honey**	1.Strážske_A	1.33	2.14	n.d.	1.97	34.90	8.80	n.d.	4.60	32.23	23.12	n.d.	n.d.	n.d.	n.d.	n.d.	1.66	0.46	n.d.	1.54	2.37
	2. Strážske_B	0.73	2.06	n.d.	24.01	67.17	32.10	n.d.	5.37	n.d.	46.12	n.d.	n.d.	n.d.	n.d.	n.d.	10.16	1.03	n.d.	4.17	n.d.
	3.Poša	1.28	1.38	n.d.	3.87	32.23	11.46	n.d.	18.49	n.d.	157.89	n.d.	n.d.	n.d.	5.36	n.d.	4.45	1.41	n.d.	2.24	n.d.
	4.Sedliska	1.34	5.05	n.d.	4.55	33.86	41.42	n.d.	7.89	25.93	60.80	n.d.	n.d.	n.d.	n.d.	n.d.	13.07	4.50	n.d.	2.40	n.d.
	5.Prešov	0.91	3.46	n.d.	3.67	149.11	54.41	n.d.	4.68	n.d.	37.19	n.d.	n.d.	n.d.	n.d.	n.d.	0.80	4.57	n.d.	2.04	9.86
	6.Košice	1.49	8.78	n.d.	14.90	46.02	50.91	n.d.	16.77	28.45	46.28	n.d.	n.d.	n.d.	9.89	n.d.	12.03	26.40	n.d.	2.69	9.81
	7.Kurima																				
**larvae/ honey**	1.Strážske_A	1.65	1.19	n.d.	1.92	13.77	5.99	n.d.	4.11	45.26	42.13	n.d.	n.d.	n.d.	n.d.	n.d.	0.58	0.12	n.d.	0.74	0.66

	2. Strážske_B																				
	3.Poša	1.14	0.98	n.d.	2.33	8.59	8.78	n.d.	26.16	n.d.	162.70	n.d.	n.d.	n.d.	n.d.	n.d.	1.51	0.55	n.d.	1.30	n.d.
	4.Sedliska	1.80	2.87	n.d.	17.59	13.78	1127.50	n.d.	11.93	46.65	158.08	n.d.	n.d.	n.d.	n.d.	n.d.	21.12	1.27	n.d.	1.29	n.d.
	5.Prešov	1.05	1.71	n.d.	2.14	7.18	17.96	n.d.	5.91	n.d.	36.47	n.d.	n.d.	n.d.	n.d.	n.d.	1.78	0.60	n.d.	1.00	2.59
	6.Košice	1.27	0.83	n.d.	1.66	4.10	9.88	n.d.	3.03	14.75	36.80	n.d.	n.d.	n.d.	n.d.	n.d.	2.51	0.64	n.d.	1.24	1.06
	7.Kurima																				
**bees/ pollen**	1.Strážske_A																				
	2. Strážske_B																				
	3.Poša																				
	4.Sedliska	0.21	0.65	0.48	1.55	0.73	1.14	1.69	0.38	0.90	0.91	n.d.	0.32	0.27	0.42	0.76	2.23	3.72	0.42	1.27	1.52
	5.Prešov																				
	6.Košice	0.19	0.84	0.81	2.04	0.74	0.90	0.81	1.54	0.98	0.69	1.15	0.90	1.04	7.11	0.41	2.80	15.77	0.59	1.39	2.02

	7.Kurima	0.36	0.38	0.38	1.23	0.24	0.87	0.49	0.17	1.34	1.34	0.99	0.35	0.77	0.51	0.25	0.65	1.29	0.28	1.15	0.87
**larvae/ pollen**	1.Strážske_A																				
	2. Strážske_B																				
	3.Poša																				
	4.Sedliska	0.29	0.37	n.d.	5.98	0.30	31.08	0.74	0.57	1.61	2.36	n.d.	n.d.	0.33	0.16	n.d.	3.61	1.05	0.29	0.69	0.55
	5.Prešov																				
	6.Košice	0.16	0.08	n.d.	0.23	0.07	0.17	n.d.	0.28	0.51	0.55	n.d.	n.d.	0.38	n.d.	n.d.	0.58	0.38	n.d.	0.64	0.22
	7.Kurima	0.38	0.32	n.d.	0.42	0.16	0.44	n.d.	0.07	1.02	1.23	n.d.	n.d.	0.99	0.47	0.10	0.54	0.02	0.19	0.89	0.41
**bees/ larvae**	1.Strážske_A	0.81	1.80	n.d.	1.03	2.53	1.47	n.d.	1.12	0.71	0.55	n.d.	n.d.	1.01	n.d.	n.d.	2.83	3.70	2.12	2.07	3.57
	2. Strážske_B			n.d.																	
	3.Poša	1.12	1.40	n.d.	1.66	3.75	1.31	n.d.	0.71	1.16	0.97	n.d.	n.d.	0.90	n.d.	n.d.	2.95	2.58	1.56	1.72	2.15
	4.Sedliska	0.74	1.76	n.d.	0.26	2.46	0.04	2.29	0.66	0.56	0.38	0.49	n.d.	0.82	2.63	n.d.	0.62	3.54	1.44	1.85	2.77

5.Prešov	0.87	2.03	n.d.	1.71	20.78	3.03	n.d.	0.79	0.85	1.02	n.d.	n.d.	0.94	n.d.	n.d.	0.45	7.68	n.d.	2.04	3.80
6.Košice	1.17	10.62	n.d.	8.96	11.22	5.15	n.d.	5.53	1.93	1.26	n.d.	n.d.	2.73	n.d.	n.d.	4.80	41.28	n.d.	2.16	9.30
7.Kurima	0.94	1.18	0.97	2.92	1.52	1.99	n.d.	2.31	1.31	1.09	n.d.	n.d.	0.77	1.08	2.64	1.21	59.30	1.48	1.30	2.13

n.d.: non-determined

Table S2: Correlation values between relevant bee samples (essential macro elements)

Sample	sampling location	Na	Mg	P	K	Ca
bees/ honey	1.Strážske_A	n.d.	n.d.	39.21	9.52	2.85
	2. Strážske_B	n.d.	n.d.	32.57	11.66	1.82
	3.Poša	n.d.	n.d.	74.90	18.61	2.85
	4.Sedliska	n.d.	n.d.	59.47	5.79	3.88
	5.Prešov	n.d.	n.d.	55.72	4.94	2.74
	6.Košice	n.d.	n.d.	76.90	4.31	5.27
	7.Kurima					
larvae/ honey	1.Strážske_A	n.d.	n.d.	93.82	27.84	2.47
	2. Strážske_B					
	3.Poša	n.d.	n.d.	152.36	46.04	2.17
	4.Sedliska	n.d.	n.d.	121.24	14.98	3.02
	5.Prešov	n.d.	n.d.	85.83	9.52	1.78
	6.Košice	n.d.	n.d.	75.46	5.41	1.29
	7.Kurima					
bees/ pollen	1.Strážske_A					
	2. Strážske_B					
	3.Poša					
	4.Sedliska	n.d.	0.35	0.54	0.62	0.30
	5.Prešov					
	6.Košice	n.d.	0.49	0.62	0.68	0.47
	7.Kurima	n.d.	0.58	0.96	0.87	0.42
larvae/ pollen	1.Strážske_A					
	2. Strážske_B					
	3.Poša					
	4.Sedliska	n.d.	0.63	1.09	1.61	0.24
	5.Prešov					
	6.Košice	n.d.	0.45	0.61	0.86	0.12
	7.Kurima	n.d.	0.60	0.95	1.06	0.26
bees/ larvae	1.Strážske_A	1.18	0.44	0.42	0.34	1.16
	2. Strážske_B					
	3.Poša	1.08	0.57	0.49	0.40	1.31
	4.Sedliska	0.85	0.55	0.49	0.39	1.29
	5.Prešov	0.65	0.60	0.65	0.52	1.54
	6.Košice	1.48	1.08	1.02	0.80	4.07
	7.Kurima	2.06	0.96	1.01	0.82	1.60

n.d.: non-determined

Table S3. Overview of computed *P*-values for Hg in Bees and Larvae

Variable\Test	Shapiro-Wilk	Anderson-Darling	Lilliefors	Jarque-Bera
Bees	0.227	0.200	0.183	0.737
Larvae	0.160	0.182	0.260	0.665

Table S4. Overview of computed *P*-values for Pb in Bees and Larvae

Variable\Test	Shapiro-Wilk	Anderson-Darling	Lilliefors	Jarque-Bera
Bees	**0.002**	**0.004**	**0.003**	0.220
Larvae	**0.016**	**0.015**	**0.008**	0.604

Table S5. Eigenvectors, factor loadings, correlations between variables and factors, and squared cosines of the observations, for toxic trace elements on bees

Eigenvectors:

	F1	F2	F3	F4
As	0.640	0.018	-0.085	-0.764
Cd	0.556	0.258	-0.580	0.537
Hg	-0.082	0.955	0.275	-0.077
Pb	0.524	-0.146	0.762	0.351

Factor loadings:

	F1	F2	F3	F4
As	0.986	0.018	-0.063	-0.152
Cd	0.857	0.262	-0.431	0.107
Hg	-0.126	0.971	0.204	-0.015
Pb	0.808	-0.148	0.566	0.070

Correlations between variables and factors:

	F1	F2	F3	F4
As	0.986	0.018	-0.063	-0.152
Cd	0.857	0.262	-0.431	0.107
Hg	-0.126	0.971	0.204	-0.015

Pb	0.808	-0.148	0.566	0.070

Squared cosines of the observations:

	F1	F2	F3	F4
Area 1	0.287	**0.662**	0.051	0.000
Area 2	**0.617**	0.102	0.281	0.000
Area 3	**0.820**	0.013	0.166	0.001
Area 4	**0.497**	0.398	0.031	0.074
Area 5	**0.683**	0.267	0.023	0.027
Area 6	0.165	**0.675**	0.024	0.136
Area 7	**0.719**	0.236	0.012	0.033

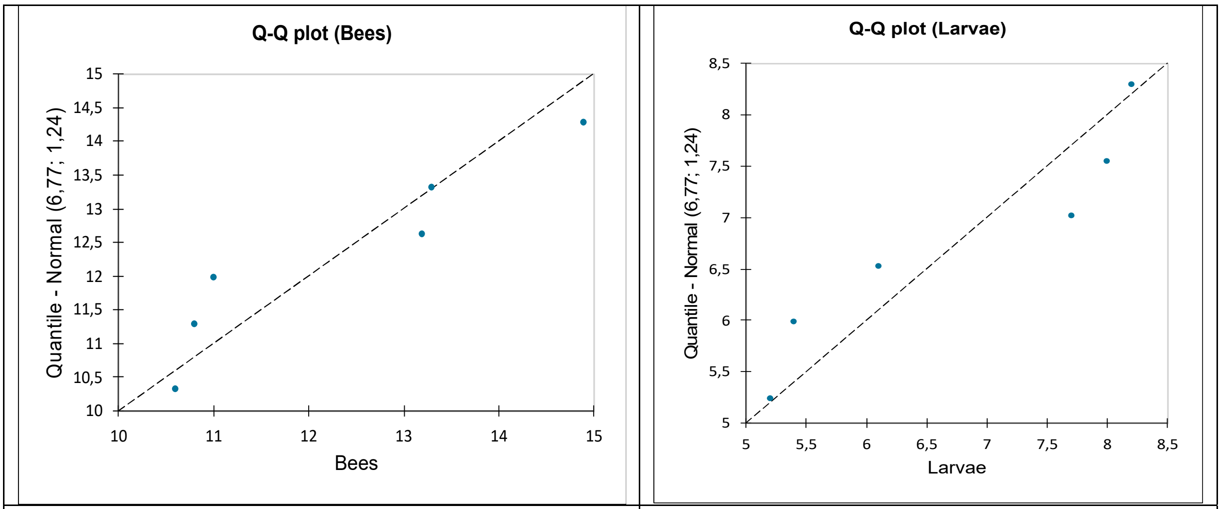

(A) (B)

Figure S1A–B. Quantile-quantile plots for Hg concentration values in bees and larvae. (A) Q-Q plot for Hg in Bees. (B) Q-Q plot for Hg in Larvae

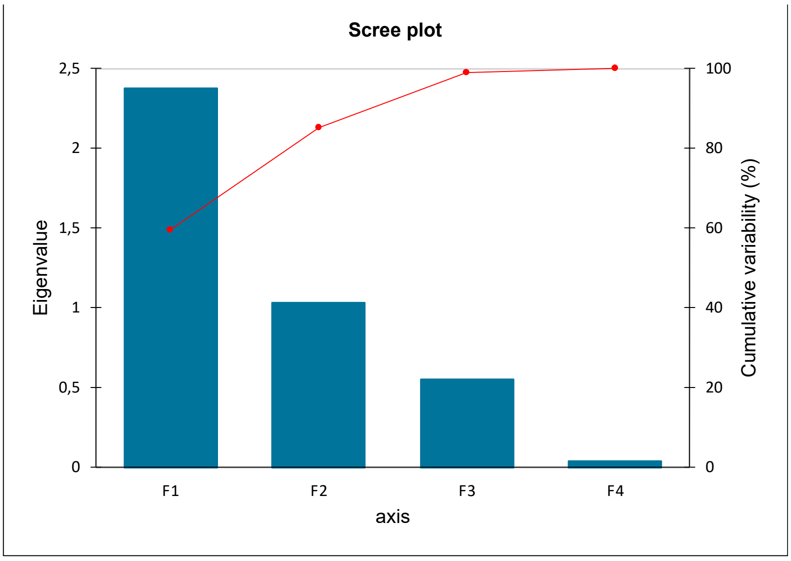

Figure S2. Scree plot of eigenvalue and % cumulative capacity for toxic trace elements on bees
